# Automatic Quantification of Serial PET/CT Images for Pediatric Hodgkin Lymphoma Patients Using a Longitudinally-Aware Segmentation Network

**Published:** 2024-04-12

**Authors:** Xin Tie, Muheon Shin, Changhee Lee, Scott B. Perlman, Zachary Huemann, Amy J. Weisman, Sharon M. Castellino, Kara M. Kelly, Kathleen M. McCarten, Adina L. Alazraki, Junjie Hu, Steve Y. Cho, Tyler J. Bradshaw

**Affiliations:** 1 Department of Radiology, University of Wisconsin, Madison, WI, USA; 2 Department of Medical Physics, University of Wisconsin, Madison, WI, USA; 3 University of Wisconsin Carbone Comprehensive Cancer Center, Madison, WI, USA; 4 Department of Pediatrics, Emory University School of Medicine, Atlanta, GA, USA; 5 Aflac Cancer and Blood Disorders Center, Children’s Healthcare of Atlanta, Atlanta, GA, USA; 6 Department of Pediatric Oncology, Roswell Park Comprehensive Cancer Center, Buffalo, NY, USA.; 7 Department of Pediatrics, University at Buffalo Jacobs School of Medicine and Biomedical Sciences, Buffalo, NY, USA; 8 Pediatric Radiology, Imaging and Radiation Oncology Core Rhode Island, Lincoln, RI, USA; 9 Department of Radiology, Emory University School of Medicine and Children’s Healthcare of Atlanta, Atlanta, GA, USA; 10 Department of Biostatistics and Medical Informatics, University of Wisconsin, Madison, WI, USA; 11 Department of Computer Science, School of Computer, University of Wisconsin, Madison, WI, USA

**Keywords:** Quantitative PET, Longitudinal Analysis, Deep Learning, Image Segmentation

## Abstract

**Purpose:**

Automatic quantification of longitudinal changes in PET scans for lymphoma patients has proven challenging, as residual disease in interim-therapy scans is often subtle and difficult to detect. Our goal was to develop a longitudinally-aware segmentation network (LAS-Net) that can quantify serial PET/CT images for pediatric Hodgkin lymphoma patients.

**Materials and Methods:**

This retrospective study included baseline (PET1) and interim (PET2) PET/CT images from 297 patients enrolled in two Children’s Oncology Group clinical trials (AHOD1331 and AHOD0831). LAS-Net incorporates longitudinal cross-attention, allowing relevant features from PET1 to inform the analysis of PET2. Model performance was evaluated using Dice coefficients for PET1 and detection F1 scores for PET2. Additionally, we extracted and compared quantitative PET metrics, including metabolic tumor volume (MTV) and total lesion glycolysis (TLG) in PET1, as well as qPET and ΔSUVmax in PET2, against physician measurements. We quantified their agreement using Spearman’s *ρ* correlations and employed bootstrap resampling for statistical analysis.

**Results:**

LAS-Net detected residual lymphoma in PET2 with an F1 score of 0.606 (precision/recall: 0.615/0.600), outperforming all comparator methods (P<0.01). For baseline segmentation, LAS-Net achieved a mean Dice score of 0.772. In PET quantification, LAS-Net’s measurements of qPET, ΔSUVmax, MTV and TLG were strongly correlated with physician measurements, with Spearman’s *ρ* of 0.78, 0.80, 0.93 and 0.96, respectively. The performance remained high, with a slight decrease, in an external testing cohort.

**Conclusion:**

LAS-Net achieved high performance in quantifying PET metrics across serial scans, highlighting the value of longitudinal awareness in evaluating multi-time-point imaging datasets.

## 1 Introduction

Among pediatric cancers, Hodgkin lymphoma (HL) is a highly curable malignancy (1), with 5-year survival exceeding 90% for patients receiving combination chemotherapy, radiation, or combined treatment (2). Despite this, pediatric patients face a significant risk of long-term side effects from therapeutic toxicities. Emerging evidence suggests that early responders to treatment may benefit from de-escalated therapies (3). Several clinical trials have used response assessment on interim Fluorodeoxyglucose (18F-FDG) PET scans after two cycles of chemotherapy for risk stratification (2,4). Currently, PET response is assessed using visual evaluation criteria, such as Deauville scores (DSs) (5). Compared to the qualitative assessment, quantitative PET metrics have shown promise in guiding lymphoma treatment strategies (6,7). However, its use often relies on manual lesion segmentation, which is difficult and time-consuming, and has been limited to clinical trial settings. Deep learning (DL) algorithms have the potential to overcome this limitation and enable automatic PET analysis.

There have been extensive studies using DL to segment lymphoma (8–11) and extract quantitative metrics (12–14) in PET scans. However, existing algorithms focus on quantifying baseline tumor burden, overlooking the important role of interim PET in response assessment. Compared to baseline PET, analyzing interim PET poses significant challenges, as tumor uptake is often subtle and difficult to differentiate from confounding physiologic or inflammatory FDG activity. Physicians typically rely on cross comparison with baseline PET to identify residual lymphoma, but methods for incorporating this information to interim PET analysis remain underexplored.

In this study, we aimed to develop a longitudinally-aware segmentation network (LAS-Net) for automatic quantification of serial PET/CT images, facilitating PET-adaptive therapy for pediatric HL patients. Central to our design is a dual-branch architecture: one branch dedicated to segmenting lymphoma in baseline PET, while the other detects residual lymphoma in interim PET. The model was trained using PET/CT images from multiple centers as part of a phase 3 clinical trial. To assess the performance of our method, we evaluated its detection performance in interim PET and its segmentation performance in baseline PET. Furthermore, we extracted various quantitative PET metrics and quantified their agreement with physician measurements. We compared LAS-Net to other methods, including those with and without the integration of baseline PET information. Lastly, we performed external testing using data from another multi-center clinical trial of pediatric HL.

## 2 Materials and Methods

### 2.1 Patient Cohort

This retrospective study included patients from two Children’s Oncology Group (COG) clinical trials: AHOD1331 (ClinicalTrials.gov number, NCT02166463) (2) and AHOD0831 (NCT01026220) (4). Both are phase 3 trials of pediatric patients aged 2–21 diagnosed with high-risk HL. The AHOD1331 trial assessed the utility of incorporating Brentuximab Vedotin with chemotherapy while the AHOD0831 trial evaluated the effects of combination chemotherapy together with radiation therapy. Baseline and interim FDG PET/CT images were gathered and transferred from IROC Rhode Island to our institution under data use agreements. Retrospective analysis was approved by institutional review board with no requirement of additional consent from patients. Of the 600 patients enrolled in the AHOD1331 trial, 200 with complete PET/CT datasets were randomly selected and used as our internal cohort. Among the 166 patients from the AHOD0831 trial, 97 had complete PET/CT datasets, and these were used for external testing.

### 2.2 Data Labeling

For the AHOD1331 dataset, three experienced nuclear medicine (NM) physicians provided lesion-level annotations for both baseline and interim PET using a semi-automated workflow (LesionID, MIM Software, Cleveland, Ohio), following a multi-reader adjudication process. One physician (M.S.) labelled all 200 cases while the other physicians (S.B.P. and S.Y.C.) each adjudicated 100 of the cases, refining the annotations by adding, deleting, or modifying contours as necessary. All segmented lesions were labeled according to physician confidence (non-equivocal or equivocal). Annotators were trained using a labeling guide (described in Appendix S1).

For the AHOD0831 dataset, PET images for each patient were annotated by one of two NM physicians (J.K. and I.L.) on Mirada XD (Oxford, UK) software as part of a prior research study (12,15). Table 1 summarizes the characteristics of these two datasets.

### 2.3 LAS-Net Architecture

**Figure 1:**
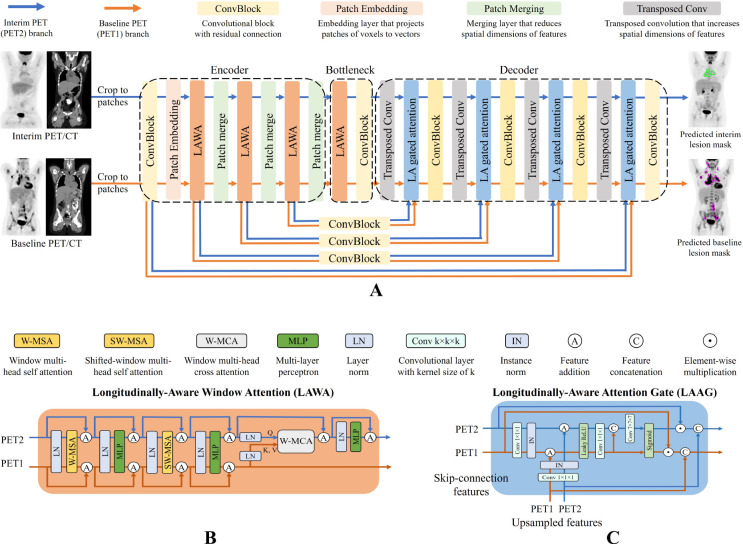
The architecture of longitudinally-aware segmentation network (LAS-Net). (**A**) The dual-branch design accommodates baseline (PET1) and interim (PET2) PET/CT images. One branch is dedicated to processing PET1 while the other branch focuses on PET2, using features extracted from PET2 as well as the features from the PET1 branch. (**B**) The longitudinally-aware window attention (LAWA) module introduces multi-head cross-attention following two self-attention blocks. All attention layers have a window size of 7. (**C**) The longitudinally-aware attention gate (LAAG) introduces a learnable convolutional layer (kernel size=7) following the standard self-attention gate to refine the attention coefficients for PET2. Both LAWA and LAAG modules only allow one-way information flow from the PET1 to the PET2 branch.

We designed LAS-Net with a dual-branch architecture to accommodate baseline and interim PET/CT images, as illustrated in Figure 1A. One branch exclusively processes baseline PET (PET1) and predicts the corresponding lesion masks. The other branch focuses on interim PET (PET2), but also utilizes information extracted from the PET1 branch to generate masks of residual lymphoma. This architecture enables our model to gather useful information from PET1 to inform and improve the analysis of subsequent scans. Meanwhile, it ensures a one-way information flow, preventing PET2 information from influencing PET1 analysis.

Like many segmentation networks, LAS-Net was adapted from a UNet-like architecture. It is based on 3D SwinUNETR (16), a state-of-the-art (SOTA) model comprising a Swin Transformer (17) encoder and a convolutional neural network (CNN) decoder. In LAS-Net, each convolutional block is a stack of two convolution units (3×3×3 convolution sub-layers, instance normalization, leaky ReLU) with a residual connection. Beyond these components, we have introduced two critical mechanisms to allow information from the PET1 branch to influence the PET2 branch. One is the longitudinally-aware window attention (LAWA) on the encoder side, and the other is the longitudinally-aware attention gate (LAAG) on the decoder side.

Figure 1B illustrates the structure of the LAWA module. Compared to the standard Swin Transformer block (17), this module introduces a window-based multi-head cross-attention (W-MCA) layer with a window size of 7×7×7 in the PET2 branch. The W-MCA takes the query vectors from PET2 features and the key and value vectors from PET1 features. It computes the attention matrix of the query and key using scaled dot product, allowing the model to dynamically allocate focus based on the relevance of regions across PET1 and PET2. The value vectors are then reweighted by this attention matrix and added to input PET2 features.

Figure 1C presents the design of the LAAG module. Similar to the original attention gate (18), the LAAG module processes inputs from both the prior layer and skip connections, generating attention coefficients. To enable additional longitudinal awareness, we concatenate the attention coefficients derived from PET1 and PET2 and convolve them with a learnable 7×7×7 kernel to refine the PET2 attention coefficients. This CNN-based cross-attention gate allows the LAAG module to select PET2 features using information from the PET1 branch.

LAS-Net operates on 112×112×112 patches from co-registered baseline and interim PET/CT images. Except for the longitudinal cross-attention components, all other weights in the model are shared between the PET1 and PET2 branches. The model was jointly optimized for PET1 and PET2 lesion segmentation using a compound loss, comprised of cross-entropy and Dice loss. Models were trained and evaluated through fivefold cross-validation (N=40 in each test fold). Implementation details can be found in Appendices S2–3.

### 2.4 Quantitative PET Metrics

In baseline PET analysis, we evaluated model performance using the Dice coefficient, false positive volume (FPV), and false negative volume (FNV) per patient. The quantitative metrics computed for PET1 scans (definitions in Appendix S4) included metabolic tumor volume (MTV) (19), total lesion glycolysis (TLG) (20), maximum lesion standardized uptake value (SUVmax), maximum tumor dissemination (Dmax) (21), maximum distance between the lesion and the spleen (Dspleen) (22) and the number of lesions. Since interim PET analysis primarily involves SUVmax or SUVpeak measurements (23), accurate tumor segmentation is not needed. Consequently, for PET2 scans, we evaluated our model’s performance using detection F1 scores, precision, and recall. Lesions detected by the model that were considered as equivocal by the physicians were not counted as false positives (FPs) or true positives (TPs) in our evaluation. We also extracted quantitative PET2 metrics from model predictions, including SUVmax, percentage difference between baseline and interim SUVmax (ΔSUVmax), qPET (23), and the number of residual lesions. Notably, ΔSUVmax and qPET have been demonstrated to have predictive potential for patient prognosis (23–25). The agreement between automated PET metrics and physician measurements was quantified by Spearman’s *ρ* correlations.

### 2.5 Model Comparison

We compared the performance of LAS-Net to other models trained on our dataset, including DynUNet (26,27), SegRes-Net (28) and SwinUNETR (16). No longitudinal cross-attention was incorporated into these models’ architectures. We also evaluated Clinical Knowledge-Driven Hybrid Transformer (CKD-Trans) (29) and Spatial-Temporal Transformer (ST-Trans) (30), both of which integrated information from PET1 into PET2 analysis using cross-attention. Notably, CKD-Trans and ST-Trans were initially developed for tumor segmentation in multiparametric MRI. Table 2 summarizes key differences among these models.

Furthermore, we implemented a previous technique (15,31) that used deformable registration between PET1 and PET2 scans to reduce FPs in PET2 lesion masks. Specifically, segmentation masks predicted for PET1 are propagated to PET2 using deformable registration, and then PET2 contours that do not overlap with PET1 contours are excluded. In our work, we refer to this technique as “mask propagation through deformable registration” (MPDR). Quantitative results were reported both with and without MPDR. Additionally, we conducted ablation studies to assess the effectiveness of individual components in LAS-Net.

### 2.6 Agreement of Predicted DSs and Physician-assigned DSs

DSs serve as an internationally accepted scoring system for assessing treatment response in interim PET. Two types of thresholds (DS3-DS5 positive or DS4-DS5 positive) are typically used to categorize patients into adequate or inadequate response classes, depending on the clinical context (32). Although our model was not trained to output patient-level DSs, we can estimate DSs by converting extracted qPET values to DSs using the qPET criterion (23). This indirect method allowed for a comparison of model-predicted DSs and physician-assigned DSs. The level of agreement was quantified by the F1 score and the Kappa index.

### 2.7 Statistical Analysis

The 95% confidence intervals (CIs) for our results were derived using nonparametric bootstrap resampling (33) with 10,000 repetitive trials. The difference between two data groups was statistically significant at 0.05 when one group exceeded the other in 95% of trials.

### 2.8 Data Availability

The COG clinical trial data is archived in NCTN Data Archive. Our algorithm was implemented using the Auto3dSeg pipeline in Monai (27). The code and models are available in the open-source project: https://github.com/xtie97/LAS-Net.

## 3 Results

### 3.1 Quantitative Performance

**Figure 2:**
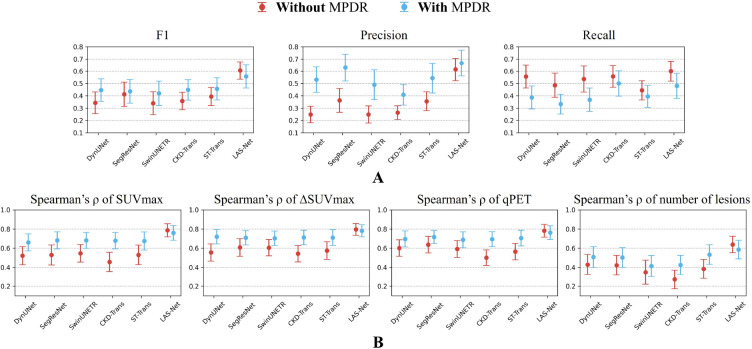
Performance comparison of interim PET lesion detection in the internal cohort. Results are reported with and without mask propagation through deformable registration (MPDR). Notably, CKD-Trans and ST-Trans utilized baseline lesion masks predicted by DynUNet for MPDR. (**A**) shows the results of evaluation metrics, including detection F1 scores, precision, and recall. (**B**) quantifies the agreement between model predictions and physician measurements for interim PET metrics. In the plots, actual metric values and Spearman’s correlation values are marked by circles with error bars indicating 95% confidence intervals. SUVmax = maximum lesion standardized uptake value, ΔSUVmax = percentage difference of SUVmax between the baseline and interim scans.

Figure 2A shows the comparison of lesion detection performance in PET2 across all evaluated models. LAS-Net detected residual lymphoma with an F1 score of 0.606 (95%CI, 0.528, 0.674). Applying MPDR to predicted interim masks increased LAS-Net’s precision (0.615 to 0.667), but at the cost of reduced recall (0.600 to 0.481). This suggests that MPDR may filter out true positive lesions, including new lesions that are not present in the baseline scan. Overall, the use of MPDR did not improve the detection performance of LAS-Net (F1 without vs. with MPDR: 0.606 vs. 0.558, P=0.22). Conversely, all comparator models benefited from MPDR, with ST-Trans (with MPDR) achieving the highest F1 score (0.446, 95%CI, 0.346, 0.538) of the comparator methods. Nevertheless, it was statistically inferior (P=0.005) to LAS-Net in identifying residual lesions. In terms of quantitative PET2 metrics, LAS-Net consistently outperformed other methods (Figure 2B), with Spearman’s *ρ* correlations of 0.79 (95%CI, 0.70, 0.86) for SUVmax, 0.80 (95%CI, 0.72 0.86) for ΔSUVmax, 0.78 (95%CI, 0.70, 0.85) for qPET and 0.64 (95%CI, 0.54, 0.72) for the number of lesions. ΔSUVmax and qPET had exact matches in values for 55% (110/200) and 69.5% (139/200) of cases, respectively.

**Figure 3:**
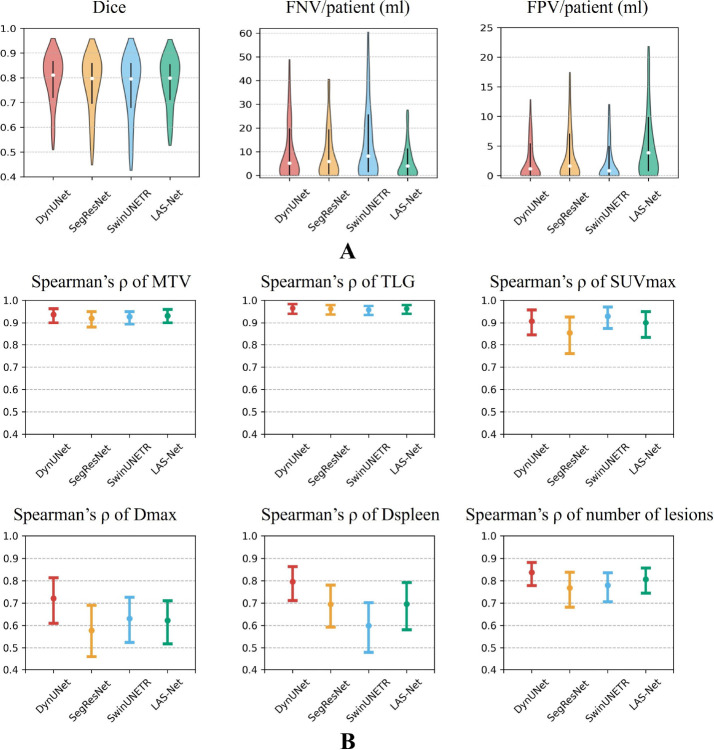
Performance comparison of baseline PET lesion segmentation in the internal cohort. (**A**) shows violin plots of evaluation metrics, where vertical lines represent the interquartile ranges and white circles mark the median values. (**B**) compares the correlations between baseline PET metrics assessed by physicians and those measured by deep learning models. Actual Spearman’s correlation values are marked by circles and their 95% confidence intervals are denoted by error bars. FPV = false positive volume, FNV = false negative volume, MTV = metabolic tumor volume, TLG = total lesion glycolysis, SUVmax = maximum lesion standardized uptake value, Dmax = maximum tumor dissemination, Dspleen = maximum distance between the lesion and the spleen.

For automatic PET1 analysis (Figure 3), LAS-Net attained a mean Dice score of 0.772 (95%CI, 0.752, 0.791), with average FNV of 10.80 ml (95%CI, 8.53, 13.46) and FPV of 9.68 ml (95%CI, 7.50, 12.40) per patient. It demonstrated comparable performance to the best model, DynUNet, which had a Dice score of 0.779 (95%CI, 0.758, 0.797, P=0.32). Among the PET1 metrics extracted by LAS-Net, MTV, TLG and SUVmax exhibited high correlations with the values measured by physicians (*ρ*=0.93 for MTV, 0.96 for TLG, 0.90 for SUVmax). No significant differences were observed across the four evaluated models for these metrics. For the distance-based metrics, Dmax and Dspleen, LAS-Net showed moderate correlations (*ρ*=0.62 for Dmax, 0.70 for Dspleen) with physician measurements, indicating the challenges of detecting individual lesions at the farthest distances.

**Figure 4:**
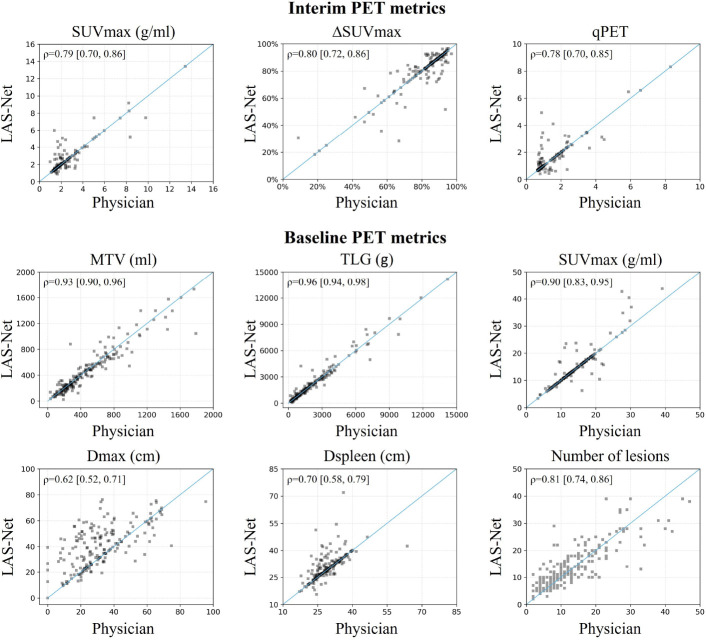
Comparison of physician-based and automatically extracted PET metrics. Spearman’s *ρ* correlations are shown in the top left corner of each plot. Correlation values are presented as mean [2.5th percentile, 97.5th percentile].

Scatter plots in Figure 4 visualize the agreement between PET metrics assessed by physicians and those measured by LAS-Net.

### 3.2 Qualitative evaluation

**Figure 5:**
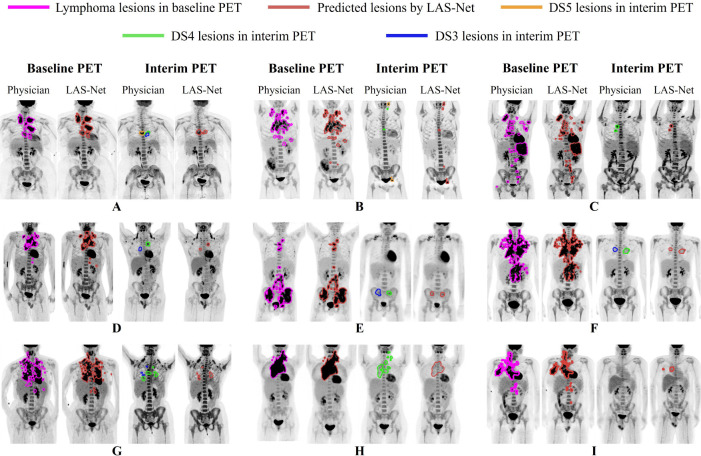
Nine different examples of longitudinally-aware segmentation network (LAS-Net) output. Each case has maximum intensity projections (MIPs) of baseline and interim PET images with overlaying MIPs of the reference and predicted lesion masks. DS = Deauville score.

Figure 5 displays images from nine sample cases, each comprising baseline and interim lesion masks predicted by LAS-Net along with physician annotations. In cases A-F, LAS-Net successfully identified the residual lesions, including the hottest lesions (DS4 or DS5) as well as those with lower uptake (DS3). Notably, in case B, LAS-Net detected new lesions, not present on PET1, located near the neck and bladder. If MPDR was applied, these true positive lesions would be excluded, leading to an underestimation of SUVmax and qPET.

In scenarios with multiple dispersed PET2 lesions (cases G-H), LAS-Net had difficulties in accurately identifying all lesions. Additionally, LAS-Net occasionally identified FP lesions in negative cases (case I), especially when the residual SUVs were close to the mediastinum uptake. For baseline lymphoma segmentation, LAS-Net performed consistently well at delineating bulky diseases. Nonetheless, it was less effective in detecting small lesions situated at a distance from the primary disease sites, which was true for all comparator methods.

**Figure 6:**
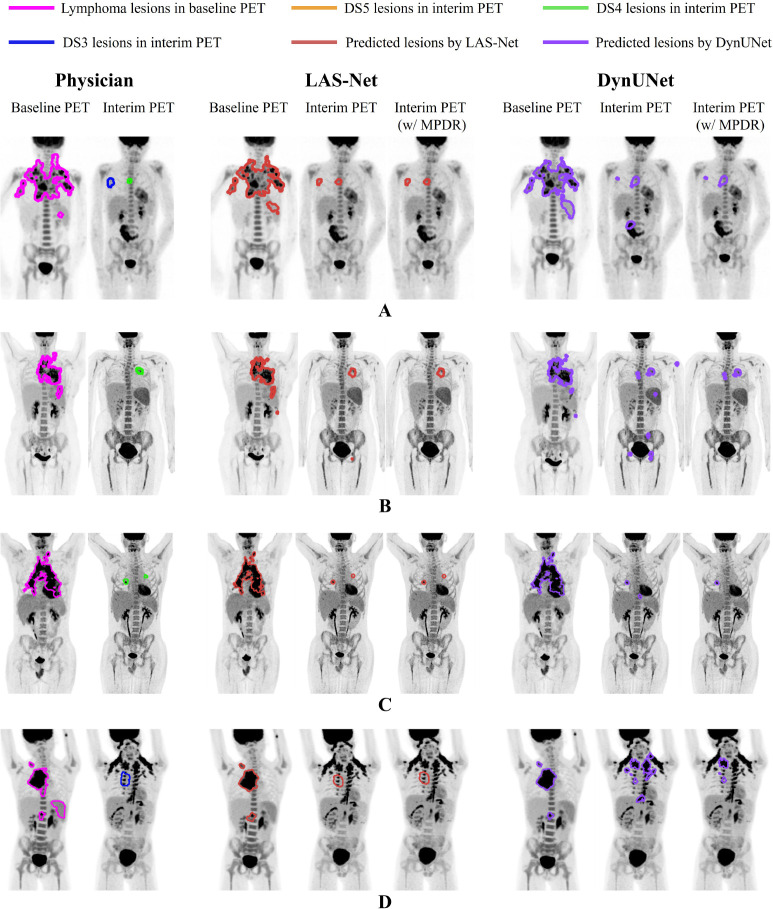
Four examples comparing the proposed longitudinally-aware segmentation network (LAS-Net) with DynUNet, a model without longitudinal cross-attention. Each case has maximum intensity projections (MIPs) of baseline and interim PET images, overlaid with MIPs of reference and predicted lesion masks. For both LAS-Net and DynUNet output, results incorporating mask propagation through deformable registration (MPDR) are also included. DS = Deauville score.

To assess the benefits of integrating longitudinal awareness into the model architecture, we compared the predictions of LAS-Net with those of DynUNet in Figure 6. Without applying MPDR, the PET2 FPs predicted by DynUNet significantly affected the accuracy of automated PET2 metrics. Especially in case D, DynUNet mistakenly identified brown fat uptake as residual lymphoma.

### 3.3 Agreement of Model-Extract DS and Physician Assigned DS

Table 3 presents DS classification results. If grouping cases into two categories – scores of DS 1, 2 vs. DS 3, 4, 5 – LAS-Net attained an F1 score of 0.752 (precision/recall: 0.687/0.836) and Cohen’s kappa of 0.630, outperforming (P<0.05) the top comparator, ST-Trans (with MPDR), which had 0.660 for F1 and 0.501 for Cohen’s kappa. If grouping based on DS of 1, 2 and 3 vs. DS 4 and 5, LAS-Net achieved an F1 score of 0.633 (precision/recall: 0.500/0.867) and Cohen’s kappa of 0.549, and was superior to other evaluated methods.

### 3.4 Ablation Studies

The results of ablation studies are shown in Table 4. We found that both LAWA and LAAG modules for longitudinal cross-attention improved lesion detection performance in PET2. Also, the inclusion of the PET1 branch and the combined PET1 and PET2 training enhanced the model’s capability to quantify PET2 scans. The choice of registration methods between PET1 and PET2 did not impact model performance. When input baseline and interim PET/CT images were co-registered using rigid registration, the performance was slightly worse but not significantly different from that achieved with deformable registration (P=0.22 for F1 scores).

### 3.5 External Testing

We applied LAS-Net, trained on all AHOD1331 data, to the external AHOD0831 dataset. The detection F1 score in PET2 was 0.525 (95%CI, 0.456, 0.582) and the Dice score in PET1 was 0.684 (95%CI, 0.655, 0.711). Regarding quantitative PET metrics, the Spearman’s *ρ* correlations between LAS-Net predictions and physician measurements showed a slight decrease: 0.70 for PET2 ΔSUVmax, 0.69 for PET2 qPET, 0.87 for PET1 MTV and 0.89 for PET1 TLG. Detailed results along with example cases are provided in Appendix S5.

## 4 Discussion

In this study, we introduced a novel deep-learning-based method (LAS-Net) for longitudinal analysis of serial PET/CT images in pediatric HL patients. Our approach was different from prior methods in two aspects. First, it used longitudinal cross-attention to extract baseline PET information for improved analysis of interim PET. Second, it adopted a dual-branch architecture to enable automatic quantification of both baseline and interim scans. Through comparative and ablation studies, we validated the effectiveness of our approach using data from two multi-center clinical trials, highlighting its potential to deliver rapid and consistent assessment of PET tumor burden and response.

Existing DL algorithms for detecting lymphoma lesions have been limited to analyzing PET1 scans without the ability to quantify PET2 for response assessment and outcome prediction. This limitation is primarily due to the challenge of detecting residual lymphoma in PET2, which often has low FDG uptake. It is even a difficult task for expert physicians, and they usually rely on PET1 (i.e., viewing PET1 and PET2 side-by-side) to identify residual lymphoma. Our method was intended to fill this gap by integrating longitudinal cross-attention mechanisms into the architecture. While previous research has leveraged prior PET data for interim image denoising (34) and response classification (35), our work distinguishes itself by incorporating longitudinal awareness to improve the analysis of multi-time-point imaging datasets.

To develop a model for longitudinal response assessment in PET scans, we chose to jointly optimize our model for PET1 and PET2 analysis. This substantially improved model performance in identifying residual lesions in PET2, as evidenced by the ablation study. However, our model’s PET1 segmentation performance was no better than other SOTA models trained on PET1 scans. This was expected, as the PET1 branch should not, in principle, benefit from the PET2 branch.

The quantitative PET metrics that we investigated have been demonstrated to be better than visual criteria at guiding lymphoma treatment (6,7). For PET1, we found that MTV and TLG were the metrics most accurately quantified by the DL model. They are also the most time-consuming metrics for physicians to measure. Newly proposed distance-based metrics (Dmax, Dspleen) were harder for accurate quantification, because a single FP or FN can have a large impact on the values of these metrics. For PET2, we focused on measuring SUVmax, qPET, and the response metric ΔSUVmax, as these have been associated with patient outcome in previous studies (23–25). Detecting residual lymphoma on PET2 was very challenging for models that did not use longitudinal information, and this was reflected in their poor F1 scores and their performance in PET2 quantification. Even with MPDR, these models were inferior to LAS-Net.

This study has several limitations. First, our training pipeline did not involve any pretraining or semi-supervised techniques. Such approaches may allow us to use unlabeled data, but whether they could enhance model performance remains to be answered. Second, we focused on quantitative PET metrics (MTV, qPET, etc.). Future research will aim to associate these metrics with patient outcome. Third, the labeling process for our external dataset differed from that used for our internal dataset. It is unclear if the performance drop in external testing is attributed to dataset shift, or different annotation quality. Fourth, our current model only operates at two imaging time points. In future work, we hope to develop a unified framework that can process PET/CT images across all time points. Lastly, we only evaluated our algorithm in the cohorts of pediatric HL patients. Whether it is applicable to other diseases or populations requires further investigation.

In conclusion, our study introduced a longitudinally-aware segmentation network to address the challenges of automatic quantification of serial PET scans. The proposed method demonstrated significantly improved lesion detection performance in interim PET without sacrificing the model’s ability to segment lymphoma in baseline PET. This technology opens opportunities to identify predictive imaging biomarkers that can lead to more effective PET-adaptive therapies.

## Supplementary Material

Supplement 1

## Figures and Tables

**Table 1:** Demographics and clinical characteristics of our internal and external cohorts.

	Internal cohort (AHOD1331)	External cohort (AHOD0831)

**Patient Characteristics**		
**Enrollment Dates**	March 2015 to August 2019	December 2009 to January 2012
**Number of Patients**	200	97
**Number of Females**	93	38
**Age (years)** **Median (range)**	15 (5–21)	16 (5–21)
**Scan Characteristics**		
**Number of PET/CT Scans**	400	194
**Injected dose (MBq)** **Median (IQR)**	314.5 (243.3, 384.3)	366.1 (279.6, 485.3)
**PET/CT scanners** **(N=number of scans)**	Siemens Biograph mCT (N=55) Siemens Biograph TruePoint (N=50) Siemens Biograph HiRes (N=29) GE Discovery ST, STE (N=92) GE Discover 600, 610, 690, 710 (N=46) GE Discovery IQ (N=12) GE Discovery RX (N=7) GE Discovery LS (N=4) GE Optima 560 (N=2) GE Discovery MI (N=1) Philips Gemini TF (N=44) Philips Allegro (N=27) Philips TruFlight Select (N=12) Philips Ingenuity TF (N=10) Philips Vereos (N=1) Philips unknown (N=8)	Siemens Biograph (N=8) Siemens Biograph TruePoint (N=25) Siemens Biograph HiRes (N=25) GE Discovery ST, STE (N = 91) GE Discover 690 (N=10) GE Discovery LS (N=26) GE Discovery RX (N=4) GE Advance (N =1) Philips Gemini TF (N =1) Philips TruFlight Select (N=1) Philips EBW NM (N=2)
**Voxel size (mm) XY, Z** **Median (IQR)**	XY: 4.06 (4.00, 4.07) Z: 4.00 (3.27, 4.00)	XY: 4.07 (3.91, 4.69) Z: 3.27 (3.27, 4.25)
**Lesion Characteristics**
**Baseline PET**		
**Total Number of lesions** *	NEQ: 2988 EQ: 140	1437
**MTV (cm3)** **Median (IQR)**	479.5 (282.8, 782.4)	391.0 (212.5, 717.9)
**Interim PET**		
**Total Number of lesions** *	NEQ: 123 (DS5: 7, DS4: 50, DS3: 66) EQ: 35 (DS5: 4, DS4: 17, DS3: 14)	149 (DS3-DS5)
**SUVmax (g/mL)** **Median (IQR)**	2.0 (1.2, 3.0)	2.2 (1.0, 4.8)

* Confidence labels (EQ and NEQ) and lesion-level Deauville scores (DS) are not available for the AHOD0831 data.

IQR = interquartile range, MTV = metabolic tumor volume, SUVmax = maximum standardized uptake volume, NEQ = non-equivocal lesions, EQ = equivocal lesions, DS = Deauville score.

**Table 2:** Characteristics of the models evaluated in this study.

Models	Model Backbone	Longitudinal Cross-attention	Using PET1 Labels for Training	Using PET2 Labels for Training	Input Scans for predicting PET1 lesion masks	Input Scans for predicting PET2 lesion masks

**DynUNet (26)** *	CNN	×	✓	✓	PET1	PET2
**SegResNet (28)**	CNN	×	✓	✓	PET1	PET2
**SwinUNETR (16)**	Transformer + CNN	×	✓	✓	PET1	PET2
**CKD-Trans (29)**	Transformer + CNN	✓	×	✓	N/A †	PET1 and PET2
**ST-Trans (30)**	Transformer + CNN	✓	×	✓	N/A †	PET1 and PET2
**LAS-Net (ours)**	Transformer + CNN	✓	✓	✓	PET1	PET1 and PET2

* DynUNet is the implementation of the nnUNet architecture in Monai.

† CKD-Trans and ST-Trans are limited to predicting PET2 lesion masks.

Note that implementations of DynUNet, SegResNet, and SwinUNETR were from Monai, whereas CKD-Trans and ST-Trans were based on the original implementations released by the authors. All models were trained and evaluated using the Auto3DSeg pipeline in Monai.

PET1 = baseline PET/CT scans, PET2 = interim PET/CT scans, CNN = Convolutional Neural Network.

**Table 3:** Results of binary classification for adequate/inadequate treatment response using model-predicted Deauville scores.

Models	DS3-DS5 positive (DS1, 2 vs. DS 3, 4, 5)		DS4-DS5 positive (DS1, 2, 3 vs. DS 4, 5)	
	
	F1	Kappa	F1	Kappa
	
**DynUNet**	0.627 [0.553, 0.695]	0.408 [0.313, 0.503]	0.469 [0.372, 0.558]	0.322 [0.231, 0.418]
**DynUNet (w/ MPDR)**	0.650 [0.566, 0.725]	0.493 [0.384, 0.597]	0.506 [0.389, 0.612]	0.393 [0.268, 0.516]
**SegResNet**	0.584 [0.500, 0.661]	0.370 [0.259, 0.478]	0.469 [0.365, 0.564]	0.332 [0.225, 0.437]
**SegResNet (w/ MPDR)**	0.618 [0.526, 0.702]	0.473 [0.358, 0.584]	0.559 † [0.434, 0.672]	0.470 † [0.334, 0.598]
**SwinUNETR**	0.576 [0.498, 0.650]	0.327 [0.228, 0.427]	0.407 [0.308, 0.497]	0.246 [0.151, 0.342]
**SwinUNETR (w/ MPDR)**	0.588 [0.497, 0.670]	0.412 [0.298, 0.521]	0.447 [0.330, 0.556]	0.325 [0.197, 0.452]
**CKD-Trans**	0.567 [0.494, 0.633]	0.265 [0.186, 0.349]	0.362 [0.271, 0.447]	0.176 [0.096, 0.258]
**CKD-Trans (w/ MPDR** * **)**	0.636 [0.560, 0.705]	0.423 [0.326, 0.520]	0.459 [0.346, 0.562]	0.327 [0.210, 0.445]
**ST-Trans**	0.580 [0.500, 0.653]	0.333 [0.234, 0.433]	0.479 [0.375, 0.573]	0.343 [0.240, 0.445]
**ST-Trans (w/ MPDR** * **)**	0.660 [0.580, 0.734]	0.501 [0.397, 0.604]	0.541 † [0.420, 0.647]	0.440 † [0.310, 0.564]
**LAS-Net (ours)**	**0.752 [0.681, 0.814]**	**0.630 [0.535, 0.718]**	**0.633 [0.523, 0.729]**	**0.549 [0.430, 0.662]**
**LAS-Net (ours) (w/ MPDR)**	0.721 † [0.644, 0.791]	0.597 † [0.497, 0.694]	0.592 † [0.471, 0.696]	0.506 † [0.372, 0.628]

* CKD-Trans and ST-Trans cannot generate lesion masks for baseline PET scans. Therefore, we used the baseline masks predicted by DynUNet for MPDR.

Data are shown as mean [2.5th percentile, 97.5th percentile]. The highest value for the given metric is highlighted in bold while † denotes values that have no statistically significant difference (P>0.05) with the best value.

MPDR = Mask Propagation through Deformable Registration, DS = Deauville score.

**Table 4:** Ablation studies evaluating the effectiveness of each component in LAS-Net for interim lesion detection.

Investigated Components	Ablation				Lesion Detection Performance in PET2		

	LAWA module	LAAG module	Using PET1 Labels for Training	Registration of inputs	F1	Spearman’s ρ of SUVmax	Spearman’s ρ of qPET
**Model architecture**	✓		✓	Deformable	0.510 [0.421, 0.591]	0.63 [0.53, 0.72]	0.68 [0.58, 0.77]
		✓	✓	Deformable	0.542 † [0.456, 0.617]	0.69 [0.59, 0.77]	0.72 † [0.63, 0.80]
			✓	N/A *	0.364 [0.267, 0.463]	0.55 [0.44, 0.64]	0.56 [0.45, 0.66]
**Necessity of PET1 branch**	✓	✓		Deformable	0.334 [0.262, 0.405]	0.49 [0.38, 0.59]	0.53 [0.43, 0.62]
**Registration method**	✓	✓	✓	Rigid	0.559 † [0.464, 0.645]	0.78 † [0.70, 0.85]	0.75 † [0.67, 0.82]
**LAS-Net (ours)**	✓	✓	✓	Deformable	**0.606 [0.528, 0.674]**	**0.79 [0.70, 0.86]**	**0.78 [0.70, 0.85]**

* Models without longitudinal cross-attention do not require co-registration between baseline and interim PET scans for training.

Data are shown as mean [2.5th percentile, 97.5th percentile]. The highest value for the given metric is highlighted in bold while † denotes values that have no statistically significant difference (P>0.05) with the best value.

LAWA = longitudinally-aware window attention, LAAG = longitudinally-aware attention gate, PET1 = baseline PET/CT scans, PET2 = interim PET/CT scans.
