# Supplementary material for: Automatic Quantification of Serial PET/CT Images for Pediatric Hodgkin Lymphoma Patients Using a Longitudinally-Aware Segmentation Network
Source: ArXiv. 2024 Apr 12:arXiv:2404.08611v1. Preprint. [Version 1] (PMC11042444)
Supplement: Supplementary file 1 [file NIHPP2404.08611v1-supplement-1.pdf]

Supplementary Material

Appendix S1. Labeling Procedures

Details of our labeling approach can be found in the labeling guide available at `https://github.com/xtie97/lymphoma_labeling_guide`. In short, lymphoma detection in the internal AHOD1331 cohort was facilitated by a customized MIM LesionID workflow. A standardized uptake value (SUV) and volume (2 ml) threshold was first used to pre-identify regions of high FDG uptake based on the PERCIST criteria (1). Then the annotator deleted regions of interest (ROIs) that did not contain tumors. Any tumor regions that were missed by pre-labelling (such as lesions $<$ 2 ml) were manually added by the annotator using the PET Edge+ tool in the MIM software. For any liver and osseous/bone marrow involvement, only focal diseases were identified. Splenic lesions were considered if focal uptake was present or diffuse uptake was higher than 1.5 of the liver SUV. When it was unclear whether a lesion was lymphoma or physiological, it was classified as "equivocal". After the first annotator labeled all cases, the second annotator (one of two senior nuclear medicine physicians) reviewed and edited the contours as necessary. Given the absence of a universally-accepted approach for delineation of tumor boundaries (2), we performed an internal calibration study to evaluate various PET thresholding methods against a set of physician-drawn contours. Consequently, we used a union of SUV $>$ 2.5 and SUV $>$ 40% of SUVmax within the lesion ROIs to create final segmentation masks. To analyze interim PET scans, the annotator compared baseline and interim PET side-by-side and used PET Edge+ to add residual tumors. Any tumor that was considered Deauville score 3-5 had an associated contour.

In the external AHOD0831 cohort, the annotator placed large ROIs around areas containing disease using Mirada XD software, excluding diffused osseous/bone marrow involvement and regions with physiological uptake. Then a union of SUV $>$ 2.5 and SUV $>$ 40% of SUVmax was applied within each lesion ROI for segmentation of the lymphoma disease. For interim PET scans, the annotator manually added the residual lesions that had FDG activity above mediastinum uptake.

Appendix S2. Image Preprocessing

In both internal and external cohorts, we resampled PET and CT images to a voxel size of 3×3×3 mm using trilinear interpolation. Labels were resampled to the same voxel size via nearest neighbor interpolation. To reduce the spatial discrepancies across longitudinal imaging data, we registered the baseline scans to the interim CT using deformable transformation. We used ANTsPy (0.4.2), a python library for medical image registration. Considering that there is a lot of background information near the edge, we cropped the PET and CT volumes using bounding boxes determined by a SUV threshold of 0.2. PET SUVs and CT Hounsfield units (HUs) were then linearly scaled from [0,30] to [0,1] and from [-150, 250] to [0,1], respectively. During training, equivocal and non-equivocal lesions were combined and used as the ground truth mask.

Appendix S3. Model Training and Inference

We first concatenated the PET and CT images as two channels for model input and then cropped random patches of 112×112×112 centered on the areas of lesion class with a probability of 0.8 (0.2 for background). To alleviate the overfitting problem, we applied the data augmentation techniques to training data, including random affine transformation (rotation between -25 and 25 degrees, axis flip for all dimensions, zoom between 0.8 to 1.2), Gaussian noise and Gaussian blur. We jointly optimized the baseline and interim PET branches, using the following loss function:

$$\begin{aligned} L(y_1, y_2, x_1, x_2; g_1, g_2) = &\Big(L_{CE}\big(y_1, g_1(x_1)\big) + L_{Dice}\big(y_1, g_1(x_1)\big)\Big) + \\ &\Big(L_{CE}\big(y_2, g_2(x_1, x_2)\big) + L_{Dice}\Big(y_2, g_2\big(x_1, x_2\big)\Big)\Big) \end{aligned}$$

Where x_1, x_2 denote baseline PET/CT (PET1) and interim PET/CT (PET2). y_1, y_2 denote reference baseline and interim lesion masks. g_1, g_2 denote the PET1 and PET2 branches of the model. Note that g_1 solely depends on PET1 while g_2 takes inputs from both PET1 and PET2. For each branch, the loss is an unweighted sum of cross-entropy (CE) loss and Dice loss, which has proven effective in various segmentation tasks (3). To enable the model to learn joint feature representations from both time points, except for the longitudinal cross-attention, all other components in the model are shared between the two branches.

The models were trained using the AdamW optimizer (4), with an initial learning rate of 10^{-4}, weight decay regularization of 10^{-5}, and a cosine annealing scheduler. We set the batch size to 3 per GPU and trained the models for 300 epochs on 2 NVIDIA A100 GPUs. The learning environment requires the following Python (3.8.8) libraries: PyTorch (1.13.0), Monai (1.3.0).

During inference, we generated lesion masks for baseline and interim PET scans separately. For baseline mask prediction, original PET1 scans were used as input for the PET1 branch, while PET2 branch inputs were set to zeros. This ensures that baseline predictions solely depend on the initial scan data. However, interim mask predictions require both PET1 and PET2 scans as input, with PET1 being deformable-registered to PET2. We employed the sliding window method with an overlap rate of 0.625 and blended outputs of overlapping patches using Gaussian weighting.

Appendix S4. Quantitative PET metrics

Table E1: Definitions of quantitative PET metrics included in this study.

Quantitative PET metrics	Definitions
**Metabolic tumor volume (MTV)**	The sum of the volumes of all suspicious lesions in the patient.
**Total lesion glycolysis (TLG)**	The product of the MTV and the average SUV (SUVmean) across all lesions
**Maximum lesion SUV (SUVmax)**	The highest SUV value measured in the hottest lesion.<br>In order to account for cases that have no residual lesions in interim PET scans, but might have false positive detections, instead of setting the SUVmax to 0 for these cases, which is unrealistic, SUVmax was set to the 95% quantile of blood pool (aorta) SUVs.
**Maximum tumor dissemination (Dmax)**	The largest distance between the centroids of any two lesions within the body.
**Maximum distance between the lesion and the spleen (Dspleen)**	The largest distance between the centroid of the spleen and all other lesions.
**ΔSUVmax**	Percentage change of SUVmax.<br>ΔSUVmax = (baseline SUVmax – interim SUVmax) / baseline SUVmax × 100%.
**qPET**	The quotient of the SUVpeak of the hottest residual lesion over the SUVmean of the liver.

Note that the aorta, liver and spleen for each patient were segmented by TotalSegmentator (5) on CT images, followed by a manual review to ensure accuracy.

Appendix S5. External Testing

Figure E1 shows the scatter plots comparing quantitative PET metrics measured by our model and by physicians in the external AHOD0831 dataset. Figure E2 presents six sample cases from the AHOD0831 dataset, each comprising model predictions and reference physician annotations.

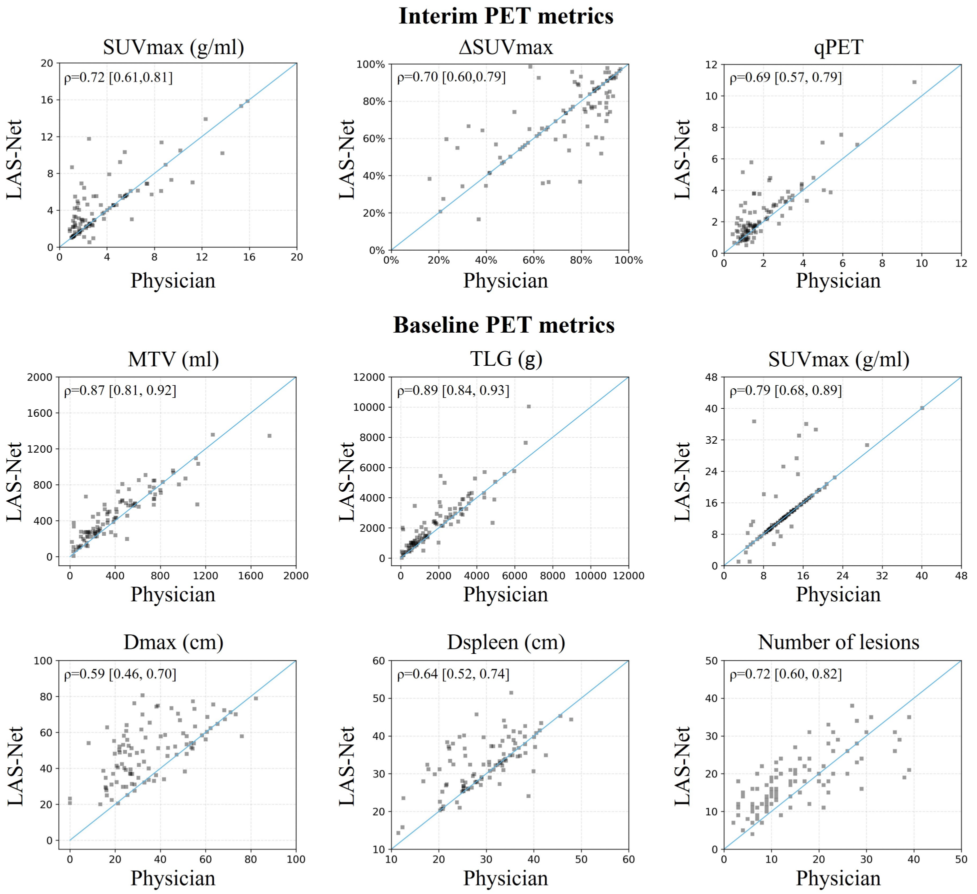

Figure E1: Comparison of physician-based and automatically extracted PET metrics in the external AHOD0831 cohort. Spearman's ρ correlations are shown in the top left corner of each plot. Correlation values are presented as mean [2.5th percentile, 97.5th percentile].

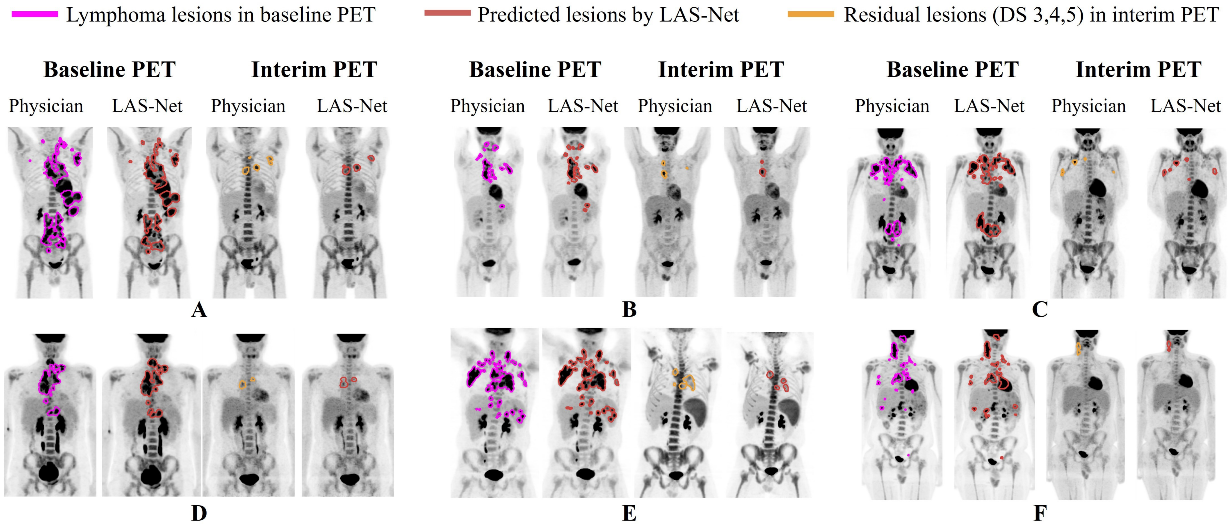

Figure E2: Six examples of longitudinally-aware segmentation network (LAS-Net) output in the external AHOD0831 cohort. Each case has maximum intensity projections (MIPs) of baseline and interim PET images with overlaying MIPs of the reference and predicted lesion masks. Note that lesion-level Deauville scores are not available for the AHOD0831 data. DS = Deauville score

References

1. Wahl RL, Jacene H, Kasamon Y, Lodge MA. From RECIST to PERCIST: Evolving Considerations for PET response criteria in solid tumors. J Nucl Med. 2009;50 Suppl 1(Suppl 1):122S-50S. doi: http://doi.org/10.2967/jnumed.108.057307.
2. Martín-Saladich Q, Reynés-Llompart G, Sabaté-Llobera A, Palomar-Muñoz A, Domingo-Domènech E, Cortés-Romera M. Comparison of different automatic methods for the delineation of the total metabolic tumor volume in I-II stage Hodgkin Lymphoma. Sci Rep. 2020;10(1):12590. doi: http://doi.org/10.1038/s41598-020-69577-9.
3. Ma J, He Y, Li F, Han L, You C, Wang B. Segment anything in medical images. Nat Commun. 2024;15(1):654. doi: http://doi.org/10.1038/s41467-024-44824-z.
4. Loshchilov I, Hutter F. Decoupled Weight Decay Regularization. arXiv; 2019. http://arxiv.org/abs/1711.05101. Accessed August 31, 2023.
5. Wasserthal J, Breit H-C, Meyer MT, et al. TotalSegmentator: Robust Segmentation of 104 Anatomic Structures in CT Images. Radiology: Artificial Intelligence. Radiological Society of North America; 2023;5(5):e230024. doi: http://doi.org/10.1148/ryai.230024.
